# Chronic rhinosinusitis and nasal polyposis in cystic fibrosis: update on diagnosis and treatment*

**DOI:** 10.1590/S1806-37132015000100009

**Published:** 2015

**Authors:** Suzie Hyeona Kang, Paulo de Tarso Roth Dalcin, Otavio Bejzman Piltcher, Raphaella de Oliveira Migliavacca

**Affiliations:** Federal University of Rio Grande do Sul, School of Medicine, Porto Alegre, Brazil. Graduate Program in Pulmonology, Federal University of Rio Grande do Sul School of Medicine, Porto Alegre, Brazil; Federal University of Rio Grande do Sul, School of Medicine, Porto Alegre, Brazil. Graduate Program in Pulmonology, Federal University of Rio Grande do Sul School of Medicine, Porto Alegre, Brazil; Federal University of Rio Grande do Sul, School of Medicine, Department of Ophthalmology and Otolaryngology, Porto Alegre, Brazil. Department of Ophthalmology and Otolaryngology, Federal University of Rio Grande do Sul School of Medicine, Porto Alegre, Brazil; Federal University of Rio Grande do Sul, School of Medicine, Department of Otolaryngology and Head & Neck Surgery, Porto Alegre, Brazil. Department of Otolaryngology and Head & Neck Surgery, Federal University of Rio Grande do Sul School of Medicine Hospital de Clínicas, Porto Alegre, Brazil

**Keywords:** Nose diseases, Cystic fibrosis, Nasal polyps, Paranasal sinuses, Sinusitis

## Abstract

Although cystic fibrosis (CF) is an irreversible genetic disease, advances in treatment have increased the life expectancy of CF patients. Upper airway involvement, which is mainly due to pathological changes in the paranasal sinuses, is prevalent in CF patients, although many are only mildly symptomatic (with few symptoms). The objective of this literature review was to discuss the pathophysiology and current therapeutic management of chronic rhinosinusitis (CRS) in CF patients. The review was based on current evidence, which was classified in accordance with the Oxford Centre for Evidence-Based Medicine criteria. When symptomatic, CRS with nasal polyps can affect quality of life and can lead to pulmonary exacerbations, given that the paranasal sinuses can be colonized with pathogenic bacteria, especially *Pseudomonas* *aeruginosa*. Infection with* P.* *aeruginosa* plays a crucial role in morbidity and mortality after lung transplantation in CF patients. Although clinical treatment of the upper airways is recommended as initial management, this recommendation is often extrapolated from studies of CRS in the general population. When sinonasal disease is refractory to noninvasive therapy, surgery is indicated. Further studies are needed in order to gain a better understanding of upper airway involvement and improve the management of CRS in CF patients, with the objective of preserving lung function and avoiding unnecessary invasive procedures.

## Abstract (pt)

A fibrose cística (FC) é uma doença genética irreversível, mas os avanços no tratamento têm aumentado a expectativa de vida dos pacientes. O acometimento das vias aéreas superiores, principalmente por alterações patológicas dos seios paranasais, é prevalente nesses pacientes, embora muitos apresentem poucos sintomas. O objetivo desta revisão é discutir a fisiopatologia e o manejo terapêutico atual da rinossinusite crônica (RSC) na FC. A revisão fundamentou-se nas evidências mais recentes, classificadas em conformidade com os critérios do *Oxford Centre for Evidence-Based Medicine*. Quando sintomática, a RSC com pólipos nasais pode afetar a qualidade de vida e as exacerbações pulmonares, já que os seios paranasais podem ser colonizados por bactérias patogênicas, principalmente a *Pseudomonas aeruginosa*. Essa bactéria tem papel crucial na morbidade e mortalidade após o transplante pulmonar em pacientes com FC. Embora o tratamento clínico das vias aéreas superiores seja indicado no manejo inicial, a indicação é muitas vezes extrapolada de estudos sobre RSC na população geral. A cirurgia é a alternativa quando o quadro nasossinusal é refratário à terapia não invasiva. Mais estudos são necessários para compreender melhor o acometimento das vias aéreas superiores e melhorar o manejo da RSC na FC, a fim de preservar a função pulmonar e evitar procedimentos invasivos desnecessários.

## Introduction

Cystic fibrosis (CF) is an autosomal recessive disease that is irreversible. It has been mapped on the long arm of chromosome 7 (7q31), which encodes the cystic fibrosis transmembrane conductance regulator (CFTR) protein. Approximately 1,000 CF-causing mutations have been identified, the most common being ΔF508.^(^ 1 ^)^ In general, CF presents as multisystem impairment, characterized by progressive lung disease, exocrine pancreatic insufficiency, liver disease, intestinal motility disorder, male infertility, and high concentrations of sweat electrolytes as a result of mucus hyperviscosity.^(^ 2 ^)^

It is well established that CF patients have upper airway involvement, and many develop chronic rhinosinusitis (CRS), which has a negative effect on their quality of life. Advances in treatment have increased the life expectancy of CF patients in recent years.^(^ 3 ^)^ This increase in life expectancy has increased the focus on the management of comorbidities, including sinonasal disease. The objective of the present review was to discuss the pathophysiology, symptoms, diagnosis, and therapeutic management of sinonasal disease in CF patients, as well as the influence of CRS on lung disease in such patients. 

## Methods

We searched the MEDLINE (PubMed), SciELO, and Cochrane Library databases using the search terms and Boolean operators (cystic fibrosis [Title]) AND (sinusites [Title/Abstract] OR paranasal sinuses [Title/Abstract] OR upper airways [Title/Abstract]) in order to identify titles and abstracts of original and review articles published between 1960 and 2013. The search was limited to articles in English, Spanish, or Portuguese. We selected the most recent and relevant articles in order to provide an update on the treatment of sinonasal disease in CF patients. 

## Criteria for the diagnosis of CRS and nasal polyposis

According to the European Position Paper on Rhinosinusitis and Nasal Polyps 2012,^(^ 4 ^)^ rhinosinusitis in adults is defined as inflammation of the nose and paranasal sinuses with two or more of the following symptoms:

- nasal congestion
- anterior or posterior nasal drip
- facial pain or pressure
- reduction or loss of smell

These symptoms can be accompanied by endoscopic signs (of nasal polyps, mucopurulent discharge primarily from the middle meatus, edema/mucosal obstruction primarily in the middle meatus, or any combination of the three), radiographic changes in the paranasal sinuses, or both. On the basis of its duration, the disease can be classified as acute/intermittent (< 12 weeks with complete resolution of symptoms) or chronic/persistent (≥ 12 weeks without complete resolution of symptoms). 

Nasal polyposis is considered to be a subgroup of CRS.^(^ 4 ^)^ In patients with CF, the finding of extensive radiographic changes in the absence of symptoms or endoscopic signs is common, its true clinical significance being unclear.^(^ 5 ^)^

## Epidemiology

Although CF has a varied phenotypic presentation, pulmonary and sinonasal involvement occurs in 90-100% of CF patients.^(^ 6 ^)^ Approximately 80% of patients with CF have nasal obstruction, 25% have anosmia, and more than 50% complain of rhinorrhea and headache.^(^ 7 ^)^ The prevalence of nasal polyposis in patients with CF appears to depend on age, increasing during adolescence and ranging from 6% to 48%.^(^ 8 ^)^

## Pathogenesis of sinonasal disease in CF patients

Although ciliary structure and beat frequency are normal in CF patients, many factors appear to contribute to impaired mucociliary clearance in such patients. First, changes in the viscoelastic properties of mucus, which are secondary to abnormal chloride conductance, appear to have a crucial role in the development of sinonasal disease. Colonization with *Pseudomonas* *aeruginosa*, which has a high affinity for the respiratory mucosa, also appears to contribute to impaired clearance. The presence of bacteria releases many substances, including homolysine and phenazine derivatives. They reduce ciliary beat frequency, and chronic inflammation causes goblet cell hyperplasia, squamous metaplasia, and hair cell loss. Macroscopically, these factors lead to sinus ostial obstruction, resulting in infected mucus stasis, local inflammation, and impaired gas exchange. Increased PaCO_2_ causes mucosal edema, decreased ciliary function, and, consequently, bacterial colonization.^(^ 1 ^)^

**Figure 1 -:**
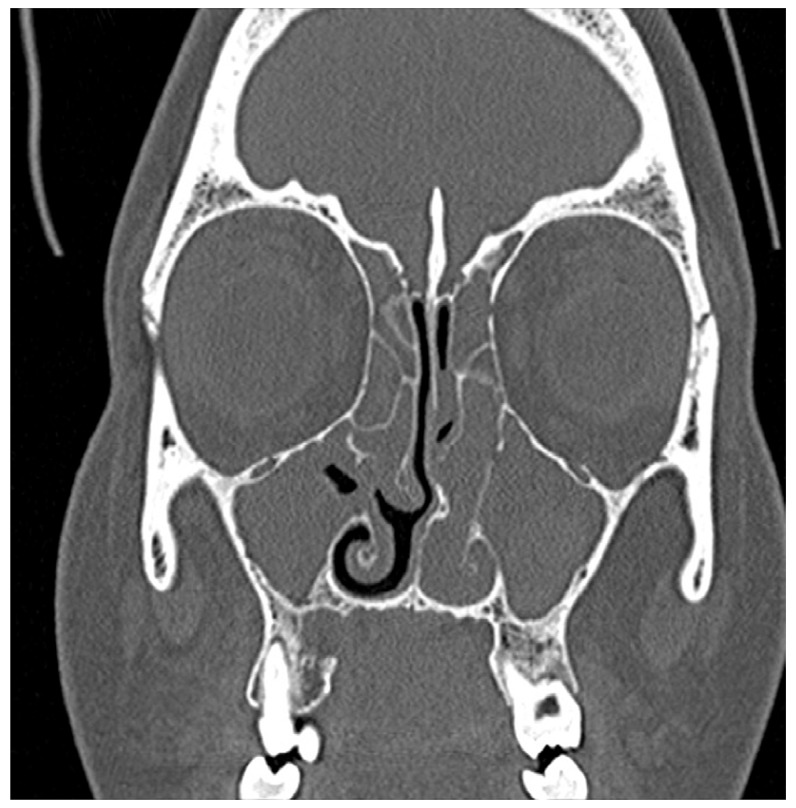
Nasal polyposis in a ΔF508 homozygous adolescent with cystic fibrosis.

It has been suggested that there is a relationship between the genotype-phenotype correlation and CRS. A high risk of nasal polyposis has been found in patients homozygous for ΔF508 or other severe mutations (Figure 1), although with no clear correlation with the severity of CF.^(^ 9 ^)^ This hypothesis was not confirmed, and some studies even showed improved lung function and nutritional status in CF patients with nasal polyposis.^(^ 10 ^,^ 11 ^)^ Genetic studies have suggested that the CFTR mutation responsible for CF is in itself a predisposing factor for sinonasal disease, showing an increased prevalence of CFTR mutations in the general population with CRS.^(^ 12 ^)^

**Figure 2 -:**
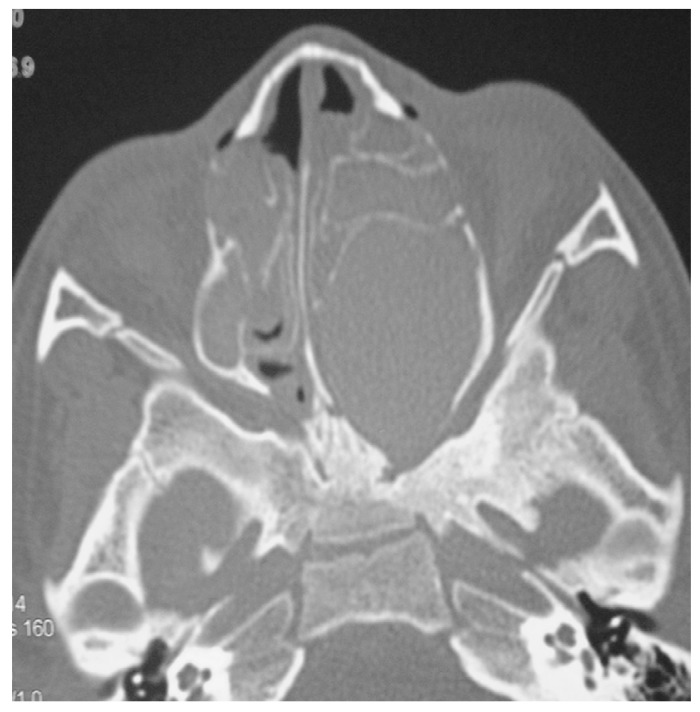
Ethmoid sinus pseudomucocele in a 6-year-old child with cystic fibrosis.

Sinonasal anatomic changes are common in patients with CF. Several factors contribute to sinus hypoplasia, including growth disorders secondary to severe chronic infections and early inflammation or changes in growth and embryogenesis caused by a genetic mutation. Erosion of the lateral nasal wall is assumed to be due to osteitis or the pressure exerted by polyps or thick mucus on the medial wall of the sinus, leading to the formation of "pseudomucocele" (Figure 2).^(^ 13 ^)^ In pediatric patients, the aforementioned changes should raise the suspicion of CF.^(^ 14 ^)^ One study showed that patients homozygous for ΔF508 were more likely to have frontal, maxillary, and sphenoid sinus hypoplasia than were those with other mutations in the CFTR protein.^(^ 14 ^)^

The reason why the prevalence of nasal polyposis is high remains unclear, as does the pathophysiology of the disease. Possible explanations include atopy and nasal obstruction causing impaired blood circulation.^(^ 15 ^)^ Studies conducted in the 1990s showed that the prevalence of atopy was no higher in CF patients than in the general population.^(^ 16 ^)^ In addition, the histopathology of CF differs from that of atopy-related polyps by the absence of eosinophilic infiltrate. It is of note that a unique etiology for nasal polyposis does not explain the pathogenesis of the disease, chronic inflammation probably being the most relevant factor.^(^ 5 ^)^

## Role of the upper airways in lung disease

In patients with CF, sinonasal involvement can exacerbate lung disease, the upper airways serving as a bacterial reservoir. Postnasal drip has been considered a major cause of lower respiratory tract infections, possibly because the bacterial florae in the paranasal sinuses and lower airways are identical.^(^ 17 ^)^

Sinus obstruction secondary to thick, impacted mucus contributes to the presence of microorganisms throughout the respiratory tree. Failure of the upper airways to filter, humidify, and warm inhaled air can be an aggravating factor in the deterioration of lung function as a result of repeated infections. This allows chronic colonization of the airways with pathogens such as *Pseudomonas* spp., which compromise airway immunity.^(^ 18 ^)^

## Sinonasal disease and lung transplantation

In lung transplant recipients, the major cause of morbidity and mortality is *P*. *aeruginosa* pneumonia, which is probably a consequence of sinonasal colonization. One study showed a significant correlation between bacterial colonization of the paranasal sinuses and lung graft infection (primarily with *P*. *aeruginosa*) after functional endoscopic sinus surgery (FESS). Patients who had undergone FESS before lung transplantation were found to have lower paranasal bacterial counts, which were correlated with reduced bacterial cultures in bronchoalveolar lavage fluid.^(^ 19 ^)^ An early study showed that daily catheter instillation of tobramycin via maxillary sinus antrostomy resulted in negative cultures for *P*. *aeruginosa*.^(^ 20 ^)^ Another comparative study, similar to the aforementioned study, showed reduced recurrence of CRS after FESS and nasal lavage with tobramycin, although no significant differences were found regarding colonization with *P*. *aeruginosa*.^(^ 17 ^)^

## Diagnosis

## History taking and physical examination

**Figure 3 -:**
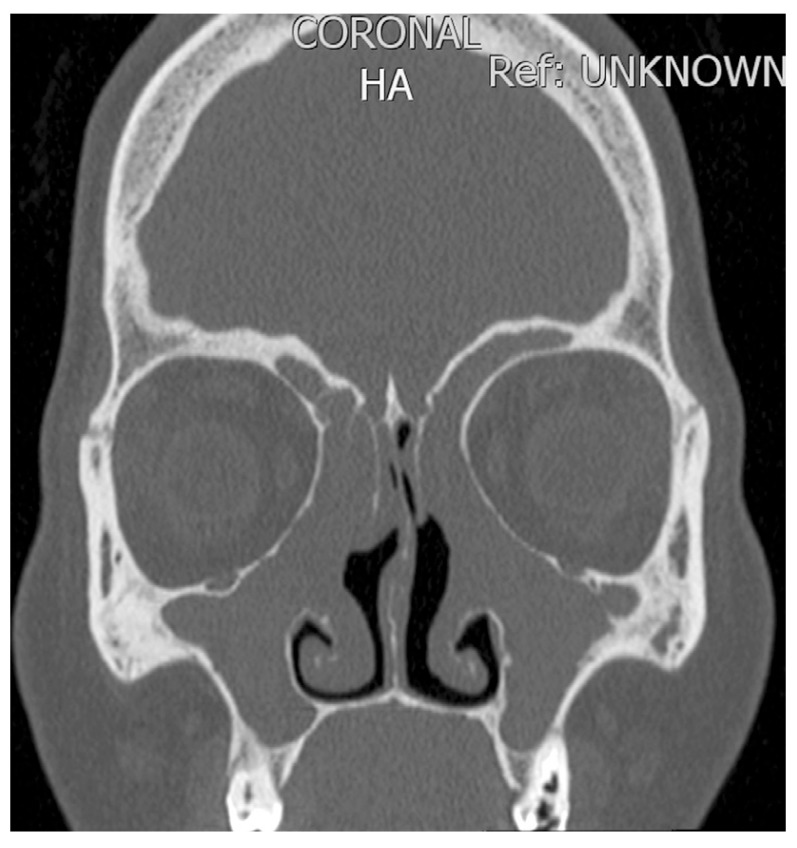
Widespread nasal polyposis and frontal sinus hypoplasia in an adult cystic fibrosis patient who had undergone nasal surgery and had no sinonasal symptoms.

**Figure 4 -:**
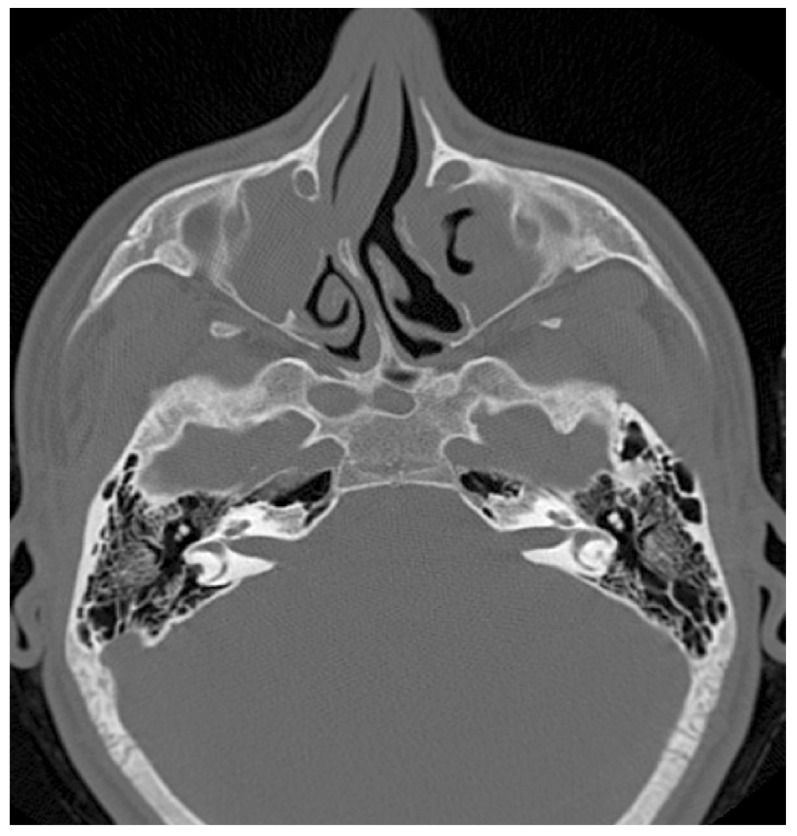
Medial bulging of the lateral nasal wall with obstructive septal deviation in an adult patient with cystic fibrosis, causing symptoms of bilateral nasal obstruction.

The symptoms of rhinosinusitis are underreported; only 10% of pediatric and adolescent patients with CF have significant complaints of sinonasal symptoms, despite changes in imaging and endoscopic findings.^(^ 7 ^)^ This adaptation to sinonasal symptoms is due to the absence of a healthy baseline for comparison. When symptoms occur, nasal polyposis (Figure 3) with consequent nasal obstruction is the main complaint. In addition, as can be seen in Figure 4, bulging of the lateral nasal wall can exacerbate the obstruction.^(^ 8 ^)^

In a retrospective study of pediatric patients, initial symptoms included nasal obstruction (in 62%), rhinorrhea (in 64%), and mouth breathing (in 38%). Chronic complaints included cough (in 60%), sleep disturbances (in 37%), headache (in 32%), and anosmia (in 12%).^(^ 7 ^)^ Headache is more prevalent in adolescents and adults, often becoming a chronic symptom.^(^ 3 ^)^

Physical examination can provide evidence of sinonasal disease, including facial deformity, broadening of the nasal root, hypertelorism, and proptosis. Anterior rhinoscopy and endoscopic examination can reveal congestion and hyperemia of the nasal mucosa, abundant secretion, polyps, and medial bulging of the lateral nasal wall.^(^ 7 ^)^

## Imaging

**Figure 5 -:**
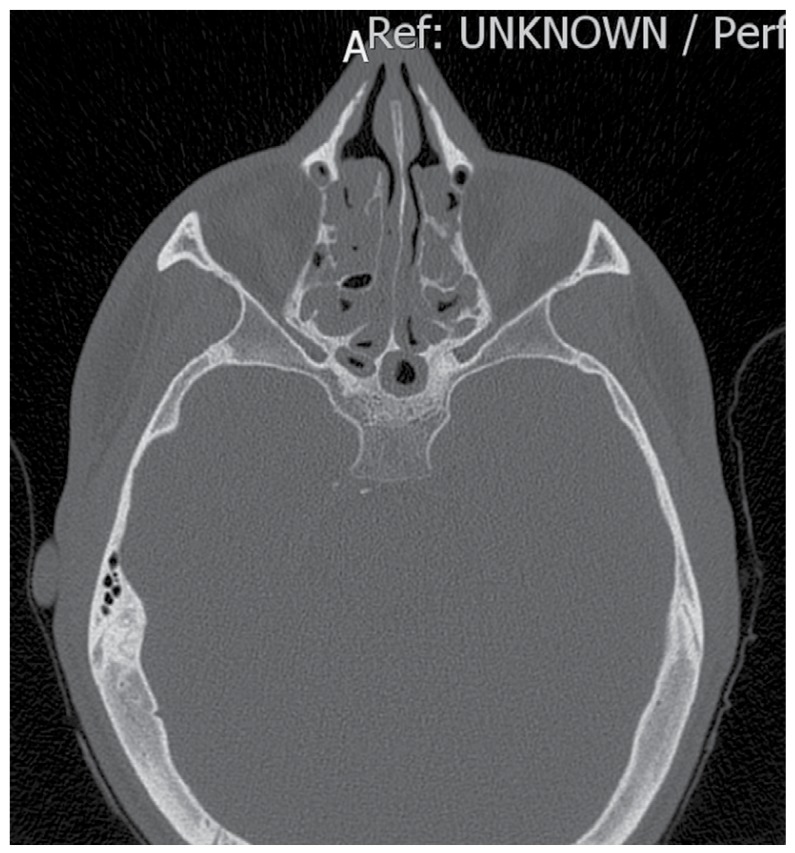
Axial CT scan of the sinuses showing sphenoid sinus hypoplasia in a 25-year-old patient with cystic fibrosis.

**Figure 6 -:**
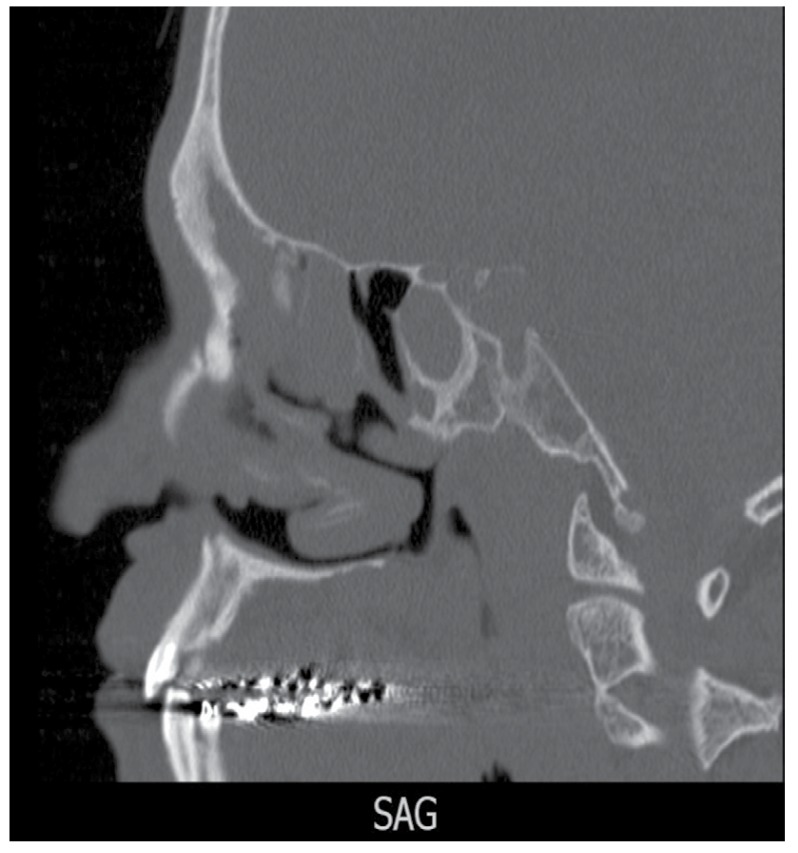
Sagittal CT scan of the sinuses showing frontal sinus aplasia in a 40-year-old patient with cystic fibrosis.

Imaging of the paranasal sinuses of CF patients reveals specific features, such as frontal and sphenoid sinus hypoplasia (Figure 5), demineralization of the uncinate process, and medial bulging of the lateral nasal wall.^(^ 21 ^)^ In most CF patients, CT imaging shows opacified paranasal sinuses after the age of 8 months. There are fewer sinus pneumatization variants, such as Haller cells and agger nasi cells. The maxillary sinuses are usually reduced in size, and the posterior ethmoid sinus usually grows faster than does the anterior ethmoid sinus, causing an inversion of their relationship throughout the ethmoid labyrinth.^(^ 7 ^)^ The presence of frontal sinus agenesis (Figure 6) and maxilloethmoid sinus opacification greater than 75% have been proposed as pathognomonic criteria for CF.^(^ 22 ^)^

CT is the gold standard imaging modality, especially for surgical planning. However, CT findings are not useful as an outcome measure for clinical or surgical interventions for CRS.^(^ 23 ^)^ Although nuclear magnetic resonance imaging allows better differentiation among mucosa, polyps, and retained secretions than does CT, it does not clearly show bony structures.^(^ 24 ^)^

## Bacteriology of the paranasal sinuses

Sputum examination and middle meatus aspiration culture are used in order to guide antibiotic therapy. The most prevalent pathogens in CF patients are *Staphylococcus* *aureus* and *P*. *aeruginosa*, the latter being the most responsible for the destruction of lung parenchyma. Pulmonary colonization with *P*. *aeruginosa* has been significantly correlated with the presence of nasal polyposis, the prevalence of which has been reported to increase with the duration of colonization with the pathogen.^(^ 25 ^)^ The phenotypic change by *P*. *aeruginosa* (to mucoid growth in macrocolonies, which inhibit phagocytosis) is the main factor for persistent airway infection, *P*. *aeruginosa* forming a biofilm and increasing its resistance, despite an intense inflammatory response.^(^ 26 ^)^ Early identification of *Pseudomonas* spp. infection is essential for the initiation of eradication therapy, the objective of which is to prevent or delay chronic infection with the bacteria at a phase in which strains are more susceptible to antibiotics.^(^ 27 ^)^

Other common bacteria in CF patients include *Haemophilus* *influenzae*, *Burkholderia* *cepacia*, *Achromobacter* *xylosoxidans*, and *Stenotrophomonas* *maltophilia*.^(^ 16 ^)^ Despite the impaired mucociliary clearance inherent to CF, patients with the disease are no more susceptible to viral upper respiratory tract infections than are those without it.^(^ 28 ^)^

Nonbacterial pathogens such as *Aspergillus* spp. are also found in sinus aspirates from more than 40% of adults with CF, a finding that signifies colonization more than it does invasive disease.^(^ 29 ^)^ In a study specifically aimed at detecting fungal sinonasal disease in CF patients, 33.3% of the cultures were positive for fungi, *Candida* *albicans* being the most frequently isolated species. Other isolated fungi included *A*. *fumigatus*, *Bipolaris* spp., *Exserohilum* spp., and *Penicillium* spp.^(^ 30 ^)^

## Clinical treatment

Conservative management is considered the first step in the treatment of CRS in CF patients. Various treatments with nasal corticosteroid sprays, decongestants, antihistamines, and saline irrigation are routinely used without a specific assessment of their efficacy. Table 1 summarizes the therapies used in CRS patients (with and without CF), the levels of evidence and grades of recommendation being based on the Oxford Centre for Evidence-Based Medicine 2011 Levels of Evidence.^(^ 31 ^)^

**Table 1 -:** Levels of evidence and grades of recommendation of studies on the treatment of chronic rhinosinusitis in patients with and without cystic fibrosis.a

Treatment	Patients without CF		Patients with CF
	CRSsNP	CRSwNP	CRS
0.9% saline nasal irrigation	Ia (A)	Ib (D)	IV (D)
3% saline nasal irrigation	Ia (A)	Ib (D)	IV (D)
7% hypertonic saline nebulization	N/A	N/A	N/A
Oral antibiotics < 4 weeks	II (B)	Ib/Ib(−)* (C)	ND
Oral antibiotics > 12 weeks	Ib (C)	III (C)	III (C)
Macrolides	Ib (C)	III (C)	III (C)
Topical nasal antibiotics	Ib(−)^b^(A−)^c^	SDD	IIb (B)
Systemic corticosteroids	IV (C)	Ia (A)	IV (D)
Nasal corticosteroids	Ia (A)	Ia (A)	Ib (A)
Recombinant human DNase	ND	ND	IIa (B)
Nasal decongestants	ND	ND	IV (D)
Leukotriene receptor antagonists	ND	Ib(−)*	ND
Ibuprofen	ND	N/A	IV (D)
FESS alone	III	III	III (B/C)

CF: cystic fibrosis; CRSsNP: chronic rhinosinusitis without nasal polyposis; CRSwNP: chronic rhinosinusitis with nasal polyposis; ND: no data; and FESS: functional endoscopic sinus surgery.

a In accordance with the Oxford Centre for Evidence-Based Medicine 2011 Levels of Evidence.(31)

b Ib(−): category Ib evidence from a study with a negative outcome.

c (A−): grade A recommendation against use.

## Nasal lavage

Normal saline (0.9% saline solution) or hypertonic saline is used in order to wash secretions, debris, and nasal crusts. Hypertonic saline has the advantage of having an osmotic decongestant effect on the nasal mucosa; however, it causes mild, reversible ciliostasis.^(^ 32 ^)^ A Cochrane meta-analysis concluded that the quality of life of patients with CRS was better with nasal lavage than without it.^(^ 33 ^)^ There are no studies of CF patients with CRS, and these recommendations are extrapolated from studies of patients without CF. Although it is advocated that 7% saline solution is more appropriate for CF patients because of its mucolytic effect, resulting in improved quality of life and reduced pulmonary exacerbations,^(^ 34 ^)^ the commercially available 3% saline solutions are the most widely used (level of evidence: IV; grade D recommendation). 

## Nasal decongestants

Nasal decongestants (oxymetazoline, phenylephrine, and xylometazoline) reduce inferior turbinate congestion but do not directly affect the maxillary and ethmoid sinuses. Rebound congestion can occur when nasal decongestants are used for more than 1 week, causing physical dependence and drug-induced rhinitis,^(^ 35 ^)^ their routine use therefore being contraindicated (level of evidence: IV; grade D recommendation). 

## Nasal corticosteroids

We found only one randomized clinical trial (RCT) of a topical nasal corticosteroid in CF patients, who received 100 μg of betamethasone nasal drops twice a day for 6 weeks. The study showed a significant reduction in polyp size. ^(^ 36 ^)^ Although CF polyps have a histological predominance of neutrophils, which theoretically do not respond to steroids, studies have shown positive effects of steroid use, which are probably due to the anti-inflammatory effect of steroids^(^ 37 ^)^ (level of evidence: Ib; grade A recommendation). 

## Oral corticosteroids

Short-term use of oral corticosteroids in the beginning of rhinosinusitis treatment with antibiotics can improve the therapeutic effects; however, this issue remains controversial. CRS nearly always coexists with lung disease, and patients receive multiple courses of antibiotics for the treatment of pulmonary exacerbations. The frequent use of antibiotics can explain the reduced incidence of rhinosinusitis complications.^(^ 38 ^)^ A Cochrane review of RCTs of oral corticosteroid use in CF patients showed slower progression of lung disease, fewer hospitalizations for respiratory exacerbations, and better quality of life but no effects on sinonasal symptoms.^(^ 39 ^)^ Although oral corticosteroids are widely recommended for patients with CRS, there is surprisingly little evidence for oral corticosteroid use, especially in CRS patients without nasal polyposis (level of evidence: IV; grade D recommendation). 

## Recombinant human DNase

Recombinant human DNase reduces the viscosity of secretions in the airways of CF patients by cleavage of extracellular DNA. One study investigated nasal inhalation of recombinant human DNase in patients undergoing FESS, showing that there was a reduction in mucosal edema more than 3 years after surgery, lower recurrence of nasal polyps, and less need for sinonasal procedures in the treatment group.^(^ 40 ^)^ One RCT showed that treatment with recombinant human DNase for 8 weeks improved nasal symptoms, as well as the CT and endoscopic appearance of the paranasal sinuses. However, the efficacy of recombinant human DNase appears to depend on surgical enlargement of the paranasal sinus ostia to allow the drug to be delivered to the sinus mucosa^(^ 35 ^)^ (level of evidence: IIa; grade B recommendation). 

## Oral antibiotics

Antibiotics constitute an integral component of the pharmacological management of rhinosinusitis in CF patients and are generally used for a period of 3-6 weeks.^(^ 16 ^)^ Although the ideal duration of treatment has yet to be defined, long-course antibiotic therapy is recommended for CF patients because of mucociliary function changes caused by abnormal ion transport and the presence of bacterial agents (*P*. *aeruginosa* and *Streptococcus* *pneumoniae*) inducing a greater reduction in mucociliary clearance.^(^ 41 ^)^

Although the choice of antibiotic therapy is empirical, the selected treatment should provide coverage for *P*. *aeruginosa*, which is one of the most common pathogens found in the lower and upper airways of CF patients. Drugs such as ciprofloxacin and azithromycin are the most widely used for prophylaxis and exacerbation control.^(^ 6 ^)^

Long-term use of azithromycin reduces airway inflammation and lung parenchymal destruction in patients colonized with *P*. *aeruginosa*, a finding that has been confirmed in RCTs.^(^ 42 ^,^ 43 ^)^ Possible mechanisms of action include direct effects on the pathogen and the host. A decrease in bacterial virulence, especially in *P*. *aeruginosa* virulence, and a late bactericidal effect, as well as a decrease in airway adherence of, motility of, and biofilm production by *Pseudomonas* spp., are the potential effects of the agent.^(^ 44 ^)^

Low doses of macrolides for prolonged periods have been used in CRS patients because macrolides play a role in modulating chronic inflammation. Macrolides are promising in the treatment of CRS because of their additional effect of reducing IL-8 production and, consequently, the size of nasal polyps (level of evidence: III; grade C recommendation).^(^ 45 ^)^

## Topical antibiotics

Tobramycin belongs to the aminoglycoside class of antibiotics, and the use of inhaled tobramycin in the treatment of chronic lower airway infections with *P*. *aeruginosa* is well established.^(^ 41 ^)^ One systematic review showed that there was insufficient evidence for the widespread use of tobramycin in patients with CRS, although tobramycin was reported to be significantly beneficial in patients with CF and CRS, especially in the postoperative management of patients undergoing FESS.^(^ 46 ^)^ Although the use of inhaled colistin and aztreonam in the treatment of the lower airways is based on strong evidence, there are no studies on the use of inhaled colistin and aztreonam in the treatment of CRS in CF patients (level of evidence: IIb; grade B recommendation).^(^ 5 ^)^

## Leukotriene receptor antagonists

Leukotrienes are inflammatory mediators found in various diseases of the respiratory tract. Patients with rhinitis and severe corticosteroid-dependent asthma with salicylate intolerance can be safely treated with leukotriene receptor antagonists (montelukast and zafirlukast). Because of their anti-inflammatory activity, leukotriene receptor antagonists are recommended for patients with CRS with nasal polyposis as an alternative to oral corticosteroids, being used in combination with topical corticosteroids. A recent meta-analysis showed a small improvement in sinonasal symptoms in patients with CRS and nasal polyposis without CF; however, the results obtained with the use of leukotriene receptor antagonists in combination with nasal corticosteroids were of no clinical relevance. ^(^ 47 ^)^ One RCT showed that montelukast reduces eosinophilic inflammation in CF patients and has positive effects on lung function, suggesting a beneficial role in preventing remodeling and bronchiolar disease.^(^ 48 ^)^ There are currently no data regarding leukotriene receptor antagonists and CRS in CF patients (no evidence available). 

## Surgical treatment

Many CF patients do not respond satisfactorily to the clinical management of CRS, 10-20% undergoing paranasal sinus surgery. Many eventually require revision surgery because of chronic sinus disease.^(^ 49 ^)^ The indication of routine FEES for the treatment of sinonasal disease in CF patients is controversial, given that the severity of its clinical presentation is subject to multifactorial influences. Patients who benefit most from surgery are those who develop recurrent disease as a result of an anatomic abnormality that obstructs sinus drainage, particularly in the presence of nasal polyposis.^(^ 42 ^)^ Several patients present with complete opacification of the maxillary sinus and normal aeration of the ethmoid sinuses. In such patients, advanced disease limited to the maxillary sinus (similar to mucoceles) might cause few symptoms, requiring no surgical treatment.^(^ 5 ^)^

Because of the chronic nature of the disease and because the primary objective of surgical treatment is symptom improvement, less invasive procedures, such as polypectomy, have been proposed. However, studies have shown that early recurrence of nasal polyps is more likely in patients undergoing polypectomy alone than in those undergoing polypectomy and procedures that are more extensive, such as intranasal ethmoidectomy and antrostomy.^(^ 50 ^,^ 51 ^)^ It has been reported that FESS should be performed in CF patients with persistent nasal obstruction after clinical treatment; in those with endoscopic or CT findings of anatomic obstruction; in those in whom there is a correlation between sinonasal symptoms and pulmonary exacerbations, especially before lung transplantation; and in those in whom symptoms such as facial pain and headache affect quality of life.^(^ 22 ^)^

One study showed a reduction in hospitalizations in CF patients in the first 6 months after FESS.^(^ 52 ^)^ A recent systematic review showed that FESS is safe in patients with CF, and that there is improvement in subjective symptoms, such as nasal obstruction, rhinorrhea, headache, facial pain, and anosmia. However, the authors found no improvement in pulmonary function test results after surgery.^(^ 53 ^)^ After that systematic review, a prospective cohort study examined the effects of FESS with adjuvant antibiotic therapy on bacterial colonization of the lower airways 1 year after surgery. The results of the study showed significantly decreased growth of pathogenic bacteria in sputum cultures after FESS, particularly in CF patients in whom sinonasal and sputum cultures were positive for the same pathogen (level of evidence: III; grade B/C recommendation).^(^ 54 ^)^

## Future research

## Intranasal gentamicin

Topical application of gentamicin appears to reduce nasal potential difference in CF patients as a result of the mechanism of correction of *CFTR* allele expression. Nasal aminoglycosides delivered via nebulization have been studied in CRS patients without CF and have been found to decrease nasal bacterial colonization and the inflammatory response.^(^ 55 ^)^

## Ibuprofen

Recent studies have described the therapeutic effects of high doses of ibuprofen in the treatment of progressive lung disease in children with CF. Although ibuprofen was found to be beneficial in a small case series of patients with CF and nasal polyposis, larger studies are needed in order to evaluate its efficacy.^(^ 56 ^)^

## CFTR modulators

New therapeutic strategies aimed at rescuing CFTR activity have been approved for selected groups of CF patients. Drugs that have undergone clinical testing include ivacaftor (VX-770), lumacaftor (VX-809), and ataluren (PTC124). Ivacaftor resulted in significant improvement in lung function in CF patients with the G551D mutation and was recently approved by the U.S. Food and Drug Administration for use in individuals who are over 6 years of age and have specific mutations.^(^ 57 ^)^ To date, there have been no studies examining the effects of CFTR modulators on the sinonasal mucosa. However, given that these new molecules attempt to "potentiate" defective chloride channels, they are assumed to have a beneficial effect on sinonasal disease in certain CF patients. 

## Gene therapy

Although gene therapy is considered the ultimate solution for CF, it is still under study. There have been gene transfer studies targeting the nasal cavities.^(^ 58 ^)^ One RCT studied the *CFTR* gene, which was transferred to the nasal mucosa via an adeno-associated virus. The method rectified abnormal nasal potential difference measurements and reduced the recurrence of rhinosinusitis during the first month.^(^ 59 ^)^

## Balloon catheter sinuplasty

Balloon catheter sinuplasty is a new therapeutic alternative for patients with CRS. It was introduced in 2006 and has been shown to be as effective as FESS. It has recently been evaluated for the treatment of CRS in pediatric patients. Balloon catheter sinuplasty has proven safe and effective and has the advantage of not involving tissue removal, sparing the mucosa.^(^ 60 ^)^ Although there have been no studies of balloon catheter sinuplasty in patients with CF, the procedure is a less invasive alternative for the treatment of CRS patients, particularly pediatric patients. 

## Final considerations

Sinonasal disease is common in children and adults with CF. In patients with CRS, the findings of noneosinophilic nasal polyps and unusual bacteria or specific radiographic findings are suggestive of CF, even in the absence of gastrointestinal or pulmonary symptoms. Children with CRS should be considered CF patients until proven otherwise and should always be screened for the disease. When symptomatic, CRS with nasal polyposis impairs the filtering function of the upper airways, contributing to the colonization of the nasal sinuses with pathogens such as *P*. *aeruginosa*. 

The pathophysiology of CF predisposes the sinonasal mucosa to chronic inflammation and recurrent infections caused by mucus stasis and anatomic changes that decrease sinus aeration. The severity of sinonasal disease can affect pulmonary status and contribute to pulmonary exacerbations. When conservative treatment does not resolve the symptoms and when sinonasal disease is related to deterioration of lung disease, FESS plays an important role. However, the diseased mucosa remains after surgery, leading to high rates of recurrence of CRS. Further studies on perioperative management are therefore required, focusing on the use of preoperative and postoperative antibiotics and anti-inflammatory agents. Such studies can help to improve the management of CRS, thus preventing recurrence and avoiding revision procedures.
